# Prospective evaluation of chronic pain associated with posterior autologous iliac crest bone graft harvest and its effect on postoperative outcome

**DOI:** 10.1186/1477-7525-7-49

**Published:** 2009-05-29

**Authors:** Carolyn E Schwartz, Julia F Martha, Paulette Kowalski, David A Wang, Rita Bode, Ling Li, David H Kim

**Affiliations:** 1 Department of Orthopedics, New England Baptist Hospital, Boston, Masschusetts, USA; 2 Departments of Medicine and Orthopaedic Surgery, Tufts School of Medicine, Boston, Massachuesetts, USA; 3 DeltaQuest Foundation, Inc., Concord, Massachuesetts, USA; 4 Department of Physical Medicine & Rehabilitation, Feinberg School of Medicine, Northwestern University, Chicago Illinois, USA

## Abstract

**Background:**

Autogenous Iliac Crest Bone Graft (ICBG) has been the "gold standard" for spinal fusion. However, bone graft harvest may lead to complications, such as chronic pain, numbness, and poor cosmesis. The long-term impact of these complications on patient function and well-being has not been established but is critical in determining the value of expensive bone graft substitutes such as recombinant bone morphogenic protein. We thus aimed to investigate the long-term complications of ICBG. Our second aim was to evaluate the psychometric properties of a new measure of ICBG morbidity that would be useful for appropriately gauging spinal surgery outcomes.

**Methods:**

Prospective study of patients undergoing spinal fusion surgery with autologous ICBG. The SF-36v2, Oswestry Disability Index, and a new 14-item follow-up questionnaire addressing persistent pain, functional limitation, and cosmesis were administered with an 83% response rate. Multiple regression analyses examined the independent effect of ICBG complications on physical and mental health and disability.

**Results:**

The study population included 170 patients with a mean age of 51.1 years (SD = 12.2) and balanced gender (48% male). Lumbar fusion patients predominated (lumbar = 148; cervical n = 22). At 3.5 years mean follow-up, 5% of patients reported being bothered by harvest site scar appearance, 24% reported harvest site numbness, and 13% reported the numbness as bothersome. Harvest site pain resulted in difficulty with household chores (19%), recreational activity (18%), walking (16%), sexual activity (16%), work activity (10%), and irritation from clothing (9%). Multivariate regression analyses revealed that persistent ICBG complications 3.5 years post-surgery were associated with significantly worse disability and showed a trend association with worse physical health, after adjusting for age, workers' compensation status, surgical site pain, and arm or leg pain. There was no association between ICBG complications and mental health in the multivariate model.

**Conclusion:**

Chronic ICBG harvest site pain and discomfort is reported by a significant percentage of patients undergoing this procedure more than three years following surgery, and these complications are associated with worse patient-reported disability. Future studies should consider employing a control group that does not include autologous bone graft harvest, e.g., a group utilizing rhBMP, to determine whether eliminating harvest-site morbidity does indeed lead to observable improvement in clinical outcome sufficient to justify the increased cost of bone graft substitutes.

## Introduction

Autologous iliac crest bone graft (ICBG) harvest is a common component of many spinal surgical procedures. Although historically considered the "gold standard" source of bone graft material, autograft bone is associated with numerous disadvantages, primarily related to additional morbidity incurred at the harvest site. Several studies have identified a surprisingly high rate of complications associated with autologous bone graft harvest, ranging from 9–49% [1-10]. The most troublesome complication has been development of acute and chronic donor site pain. In the setting of anterior cervical fusion surgery, pain associated with autograft harvest often overshadows pain from the primary surgical site [11]. A perceived high rate of donor site pain has been one of the strongest factors driving a sustained search for alternatives to autograft, such as allograft bone, ceramics, and biologics including recombinant human bone morphogenetic proteins (rhBMPs). Recent clinical trials have demonstrated excellent biological potency of rhBMPs that may even surpass the efficacy of autograft in terms of promoting spinal fusion [12]. Unfortunately, the high cost of rhBMPs suggests that appropriate initial applications for rhBMPs will be limited.

Various strategies have been applied in an effort to reduce the patient's experience of postoperative donor site pain, including administration of local anesthesia with or without narcotics, as separate injections or as an infusion [13]. Postoperative injection of long-acting anesthetic (e.g., bupivacaine hydrochloride 0.25%) reduces postoperative pain in the acute period, and addition of morphine appears to have an added benefit [14,15]. Use of an indwelling catheter to administer a continuous infusion of anesthetic in the early postoperative period does not appear to decrease donor site pain and may increase the risk of wound infection at the catheter site [16]. Local anesthesia has not been shown to reduce the rate of chronic donor site pain. Attempts have been made to reduce morbidity by alterations in the technique of ICBG harvesting [17,18]. Keeping the outer and inner cortical tables intact does not reduce the severity of pain at the donor site [19]. Closed methods of graft harvesting with cylindrical osteotomes and percutaneous needle techniques have been used successfully in craniofacial surgery to retrieve small quantities of cancellous bone graft with reduced morbidity but cannot be used for corticocancellous graft harvest and do not result in sufficient quantity of graft for most spinal applications [20,21].

Complete avoidance of autologous bone graft harvest is the only way to eliminate the significant risk of chronic donor site pain. Unfortunately, the high cost of effective alternatives such as rhBMPs prohibits widespread use; and other options such as allograft, synthetic substitutes, and non rhBMP so-called factor- or cell-based substitutes have been associated with less favorable clinical results. The purpose of this study is to investigate the long-term impact of the ICBG complications on patient function and well-being in a cohort of patients undergoing autologous ICBG harvest for various spinal procedures. We also aimed to evaluate the psychometric properties of a new measure of ICBG morbidity that would be useful for appropriately gauging spinal surgery outcomes.

## Materials and methods

This prospective single institution study involved spinal fusion patients who had undergone autologous ICBG harvest as a component of their surgery between 2003 and 2005. During the fusion procedure, a separate incision was made over the posterior iliac crest through the subcutaneous tissue down to the fascia, where subperiosteal dissection was used to expose the outer table of the ilium. A retractor was placed into the wound under direct visualization, and cortical and cancellous bone graft strips were harvested. No patients had indwelling local anesthetic infusion catheters and no patients had bone graft harvest of both tables. There were no hernias related to the bone graft harvest site. There was no measurement of bone graft volume made for each surgery. All wounds were closed in layered fashion with absorbable suture material. Local anesthetic was infiltrated into the bone graft harvest site. The patients with increased post-operative graft harvest site pain were not treated with a pre-specified protocol. They were treated on an individual basis at the discretion of the surgeon. The most common treatments included oral narcotics, nonsteroidal anti-inflammatory medication, topical anesthetic patches (i.e., Lidoderm patches), and hot/cold therapy. We were unable to discern a clear advantage in terms of one treatment modality over another, but this was not a predefined question of the study design.

We implemented the study using the Dillman Tailored Design Method [22], a detail-oriented survey implementation method that relies on empirically documented techniques for maximizing response rate. Patients provided written authorization for release of medical record data and informed consent for this study. This study was approved by the New England Baptist Hospital Institutional Review Board for the protection of human subjects (IRB Protocol  2003–006).

Outcomes were measured using the Short-Form-36v2 (SF-36v2) [23], the Oswestry Disability Index (ODI; for lumbar patients) or Neck Disability Index (NDI; for cervical patients) [24], and Visual Analogue Scale (VAS) items for surgical site pain and arm or leg pain. Additionally, two new study-specific questionnaires were used. At baseline, an 11-item questionnaire assessed pain syndrome history, tolerance to pain, and use of narcotic or anti-depressant medications. Post-operatively, a new 10-item ICBG Questionnaire assessed the presence or absence of persistent pain, functional limitation, and cosmesis at the ICBG site (see Additional file 1). We did psychometric analyses of the ICBG pre-operative and post-operative questionnaires to determine how the data should be used in subsequent analyses. T-tests and chi-squared tests compared the baseline and follow-up samples on demographic characteristics to assess possible selection bias in this study. Multivariate linear regression examined the independent effect of ICBG complications on physical and mental health, and disability, after adjusting for age and workers' compensation status, variables presumed to be associated with worse outcomes. The multivariate model also adjusted for pain at extremities and pain at surgical site to ensure that the results focused on the impact on clinical outcomes specific to ICBG-related complaints. The correlations were moderate between the ICBG Complications Score and the VAS at the primary site and extremity site (r = 0.45 and 0.37, respectively), suggesting that collinearity was not a problem in our regression models involving these variables. There was, however, an issue of multicollinearity between the VAS graft site score and post-operative ICBG summary score (r = 0.71, p < 0.0001). We thus did a principal component analysis to create a summary score of the ICBG-related complications, which included the 8 items from the post-operative ICBG questionnaire and the VAS graft site pain item (Eigenvalue = 1.71, 85% of data variance explained). This variable was then included in subsequent multivariate regression models.

## Results

One-hundred-seventy questionnaires were obtained from a total of 205 patients contacted, resulting in a response rate of 83%. Of these 170 patients, longitudinal data for both one-year and final follow-up were available on a subgroup of 139 patients. Although most analyses were done with the full sample of 170 patients, one psychometric analysis utilized the smaller sample of 139 with longitudinal data to assess the stability of the measure.

### Selection bias analyses

A comparison of the baseline and follow-up samples revealed they were comparable in age, gender distribution, diagnosis distribution, smoking status, worker's compensation status, time since surgery, and pre-operative reporting of chronic pain syndrome, severe pain, and pain tolerance (Table 1).

**Table 1:** Comparison of demographic and clinical characteristics for the baseline and follow-up study samples*†

Variable	Baseline sample (n = 205)	Follow-up sample (n = 170)	p-value of t-test or chi-square comparing the 2 samples
Age (M, sd)	50.8(12.1)	51.1(12.2)	P = 0.85
Gender (%)	51.2% female (105)	51.8% female (88)	P = 0.92
Time since surgery (sd)	1326.5 days (253.4)	1308.1 days (257.1)	P = 0.49
Procedure (cervical or lumbar)	---------	87.1% lumbar (148)	----------
Diagnosis			
% Stenosis (N)	37.2%(67)	38.9% (58)	P = 0.75
% Degenerative Disc Disease (N)	43.3% (78)	39.6% (59)	P = 0.49
Smoking status	24.9% yes (49)	22.8% yes (37)	P = 0.65
Workers compensation status	19.6% yes (40)	17.8% yes (30)	p = 0.65

Pre-op Scores			

Endorsing chronic pain syndrome (N)	12.9% yes (26)	11.5% yes (19)	P = 0.67
Endorsing regular severe pain from medical condition (N)	20.2% yes (41)	19.1% yes (32)	P = 0.78
Pain tolerance (M, sd)	6.7(2.1)	6.8(2.0)	P = 0.98

Post-op scores			

ICBG Complications score (sum of yes/no)	-----------	1.2(2.1)	----------
VAS for pain at graft site	-----------	13.1 (21.9) (0–96)	----------
VAS for pain at primary surgical site	-----------	25.7(27.5) (0–91)	----------
VAS for pain in extremities	-----------	23.2(27.5) (0–99)	----------
SF-36 Physical Component Score	-----------	40.7(11.5)	----------
SF-36 Mental Component Score	-----------	47.2(13.8)	----------
*Oswestry Disability Index*	-----------	28.6 (22.4)	----------

† Abbreviations used in this table: M = mean, sd = standard deviation, N = sample size

### Study sample

The study population included 170 patients with a mean age of 51.1 years (SD = 12.2) and balanced gender (51.8% female) (Table 1). Lumbar fusion patients predominated (cervical n = 22; lumbar = 148). At an average of 3.58 years of follow-up (range = 1.1 to 4.62 years), the sample reported physical and mental health scores below the general population (mean = 40.7 and 47.2, respectively; as compared to age- and gender-adjusted population norms of 50) and Oswestry scores that reflect moderate disability (mean = 28.6, which is in the moderate disability range as per Fairbank published criteria [24,25]).

### Psychometric analyses of ICBG Complications questionnaire

Tetrachoric correlations were used in the factor analyses to evaluate the unidimensionality of the pre- and post-operative study-specific questionnaires. Results revealed that pre-operative questions were not unidimensional and could not be scaled together. Consequently, analyses using these baseline data were done on individual items for descriptive purposes only and not used in subsequent inferential analyses. In contrast, the factor analysis of the 10-item post-operative ICBG questionnaire revealed that a two-factor model fit the data well, with all but one item loading on a single factor and showing good internal consistency (α = 0.88). We should note that the Exploratory Factor Analysis results and they differ when using tetrachoric and polychoric correlations. With polychoric, two factors are identified (scar issues and ensuing difficulties with item 1 loading weakly on F1); the first factor explains 66% of the variance and the first two factors explain 80%, suggesting two factors. With tetrachoric, one factor is identified (item 1 loading weakly) and it explains 58% of the variance. We formed the scale score on the basis of the tetrachoric because this approach is generally considered more appropriate for dichotomous items. While using tetrachoric correlations might be more appropriate, the results using polychoric appear to be what one would expect looking at the item content.

We used a criterion validity approach to evaluate the responsiveness of the ICBG, given the cross-sectional nature of the data. An analysis of variance revealed that the ICBG Complications score was significantly different by ODI score (F = 9.36, p < 0.0001), with the largest group difference being between those with very low ODI scores as compared to the remainder of the sample. To evaluate the stability of the measure, we compared ICBG Complications scores on a subgroup of 139 patients for whom both one-year and final follow-up data were available. There was no difference in ICBG Complication score between the one-year and final follow-up data points (paired sample t-test = -0.84, p = 0.40). This suggests that the measure is stable during a period when little change is expected. There may, however, be a floor effect; approximately half of the sample reported zero ICBG Complications.

An analysis of missing data patterns revealed that one item related to numbness was missing data because of a skip pattern in the questionnaire, and one item about job-related difficulty was missing data because of the number of retired patients. These were recoded to "0" or "1" as appropriate allowing retention of all patients in the analyses without changing overall relationships between ICBG morbidity and clinical outcomes. Validity of this approach was confirmed by scatter plots.

### Prevalence of ICBG Pain

**Figure 1:**
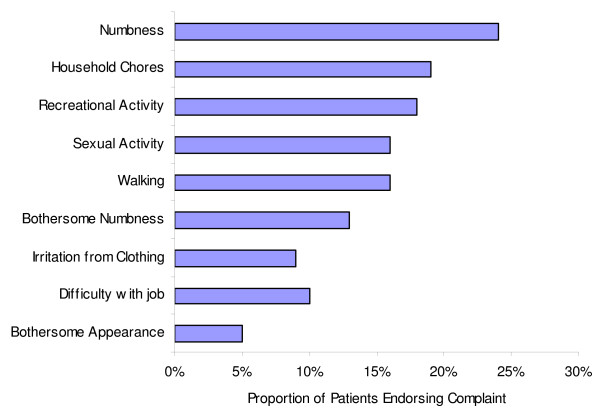
**Proportion of follow-up patients endorsing ICGB-related symptoms**. At a mean of greater than 3 years following surgery, a relatively large percentage of patients continued to report being troubled by harvest site scar appearance (5%), numbness (24%), and bothersome numbness (13%). In particular, chronic harvest site pain resulted in difficulty with household chores (19%), recreational activity (18%), walking (16%), sexual activity (16%), their job (10%), and irritation from clothing (9%).

At a mean of greater than 3 years following surgery, a relatively large percentage of patients continued to report being troubled by harvest site scar appearance (5%), numbness (24%), and bothersome numbness (13%). In particular, chronic harvest site pain resulted in difficulty with household chores (19%), recreational activity (18%), walking (16%), sexual activity (16%), their job (10%), and irritation from clothing (9%) (Figure 1).

### Regression Analyses

Regression analysis revealed that chronic ICBG harvest-related complaints were associated with significantly worse disability on the ODI 3.5 years post surgery (β = 3.5, p < 0.02, R^2 ^= 0.60), after adjusting for age, compensation status, pain at the primary surgical site, and residual extremity pain (Table 2). ICBG harvest-related complaints showed a trend relationship with worse physical functioning (β = -1.55, p < 0.07, R^2 ^= 0.40), after adjusting for age, compensation status, pain at surgical site, and pain at extremities. There was no association between ICBG harvest-related complaints and mental health (β = -1.33, p = 0.24, R^2 ^= 0.27), after adjusting for age, compensation status, pain at surgical site, and pain at extremities. Whereas age was a significant covariate of physical health, it was not related to mental health or disability. Workers' compensation status was a significant covariate of disability, but not of physical or mental health. Persistent pain at the primary surgical site and affecting the extremities were significant predictors of worse scores on all outcomes.

**Table 2:** Results of multiple linear regression models investigating impact of ICBG complications on mental and physical health and disability

Outcome	Variable	Parameter Estimate	Standard Error	t statistic	Pr > |t|	R^2^
SF-36 Mental Component Score N = 142*	age	0.05233	0.08391	0.62	0.5339	0.270
	COMPENSATION	3.65895	2.74025	1.34	0.1840	
	ICBG Complications	-1.32793	1.12835	-1.18	0.2413	
	VAS Pain at Surgical Site	-0.14489	0.04534	-3.20	0.0017	
	VAS Pain at extremities	-0.09867	0.04355	-2.27	0.0251	

SF-36 Physical Component Score N = 143	age	-0.15777	0.06213	-2.54	0.0122	0.396
	COMPENSATION	1.67623	2.02666	0.83	0.4096	
	ICBG Complications	-1.54756	0.83678	-1.85	0.0666	
	VAS Pain at Surgical Site	-0.13996	0.03366	-4.16	< .0001	
	VAS Pain at extremities	-0.11407	0.03229	-3.53	0.0006	

Oswestry Disability Index N = 140	age	0.00008492	0.09885	0.00	0.9993	0.598
	COMPENSATION	-7.66128	3.21902	-2.38	0.0187	
	ICBG Complications	3.45739	1.34204	2.58	0.0111	
	VAS Pain at Surgical Site	0.29556	0.05390	5.48	< .0001	
	VAS Pain at extremities	0.27288	0.05095	5.36	< .0001	

## Discussion

Chronic ICBG harvest site pain and discomfort is reported by a significant percentage of patients undergoing this procedure more than three years following surgery, and these complications are associated with worse patient-reported disability and somewhat worse physical health, but not with mental health. These findings suggest that morbidity associated with autologous ICBG harvesting for spinal surgery is clinically important and enduring. A significant negative impact is apparent even after controlling for persistent low back pain and extremity pain.

Despite numerous clinical studies investigating the incidence of chronic pain and morbidity associated with autologous bone graft harvest for various surgical procedures, the true magnitude of this problem remains the subject of ongoing controversy. Available studies of iliac crest bone graft harvesting have reported a wide range of complication rates from 9.4 to 49%, with minor complication rates ranging from 6 to 39% and major complication rates ranging from 0.7 to 25% [3-10]. The literature on this topic must be reviewed with caution. The reported risk of specific complications such as pain and sensory loss varies widely among studies largely due to variations in study design and differing patient populations. Nearly all studies have been retrospective in nature, which is a well-recognized problem in terms of determining true complication rates.

The largest published series to date is by Arrington et al. and consists of a retrospective chart review of 414 patients undergoing iliac crest bone graft harvest for either orthopedic or oral-maxillofacial reconstructive surgery [3]. Minor and major complication rates of 10% and 5.8%, respectively, were observed, but the authors chose to specifically exclude chronic donor site pain from the study, because they believed this data could not be accurately determined through a retrospective study design. The largest reported series to include an assessment of chronic pain is a retrospective review by Banwart et al. and included 261 mostly spine surgical patients undergoing nonstructural anterior or posterior iliac crest bone graft harvest [4]. Of 180 patients meeting requirements for statistical analysis, there were 18 (10%) complications considered major, including 3 (1.7%) patients with chronic harvest site pain limiting activity, a case of prolonged wound drainage, a seroma, and 13 cases of patients complaining of unsightly scars. Sensory changes occurred in 31%. The authors concluded that the rate of major complications is low, but minor complications occur commonly and do not appear on chart review.

Among studies that focus primarily on the occurrence of chronic harvest site pain there remains wide variability in reported rates of this complication. Based on chart review data of 239 patients, Younger and Chapman reported a minimal 2.5% rate of persistent pain at 6 months [10]. By contrast, Summers and Eisenstein reported a 49% rate of chronic donor site pain (25% severe, and 24% "acceptable") in a series of 290 patients undergoing lumbar spinal fusion surgery [9].

Only one previous study has attempted to address the functional impact of chronic harvest site pain. Silber et al. performed a retrospective questionnaire study of 134 patients undergoing anterior iliac crest bone graft harvest for anterior cervical fusion surgery and found generally high rates of chronic morbidity, including 26.1% for donor site pain, 15.7% for abnormal sensation, and surprisingly high rates of impairment with ambulation (12.7%), recreational activity (11.9%), work activity (9.7%), activities of daily living (8.2%), sexual activity (7.5%), and household chores (6.7%)[11]. Although the authors acknowledge the risk of significant bias inherent in their retrospective data, the results are remarkably consistent with the high rate of chronic pain, sensory alteration, and functional limitation found in our prospective study.

The largest previously reported prospective study of autologous bone graft harvest is by Robertson et al. and involved 106 patients undergoing posterior spinal fusion [26]. Although 55% of patients reported no pain at 12-month follow-up, 12% of patients reported a harvest site VAS pain score greater than 3 and persistent local sensory loss was reported in 10%. No attempt was made to determine whether chronic harvest site pain was associated with any specific functional limitations.

The strengths and limitations of this study should be noted. The strengths include the development of a psychometrically sound tool for evaluating ICBG morbidity. Further, this is the longest prospective study of ICBG morbidity to date with sufficient sample size to allow multivariate analyses of the independent impact of ICBG morbidity on physical and mental health. The limitations of the study are that 35 (17%) patients did not provide complete data on all of the questionnaires leading to a sample size of 170 in the multivariate regression models. This missing data problem may result in biased estimates of the effect of ICBG on clinical outcomes and may reduce our statistical power to detect clinically important effects on physical health. Second, our measures of key outcomes were limited. The pain measure was based on three individual VAS items, which have lower reliability than multi-item questionnaires. Future studies should consider employing a control group that does not include autologous bone graft harvest, e.g., a group utilizing rhBMP, to determine whether eliminating harvest-site morbidity does indeed lead to observable improvement in clinical outcome sufficient to justify the increased cost of bone graft substitutes. Finally, we had only cross-sectional data available for the full sample analysis and were thus unable to evaluate patterns of change over time. Future research should explore whether the new questionnaire used in this study can be improved by utilizing Likert rather than dichotomous response options. It would be worthwhile to investigate whether this modification in respondent options might reduce the floor effect detected in our data.

## Conclusion

In summary, the long-term impact of ICBG is substantial and clinically important. Our study suggests that about one-fifth of patients experience substantial pain and disability even three years after surgery, and these symptoms affect functioning. This information would support the use of bone graft substitutes that avoid ICBG harvesting. In addition to this clinical implication, our study provides a useful tool for the continued evaluation of ICBG complications for future research that seeks to evaluate the comparative efficacy of ICBG as compare to rhBMP.

## Abbreviations

(ICBG): Iliac Crest Bone Graft; (rhBMPs): recombinant human bone morphogenetic proteins; (SF-36v2): Short-Form-36v2; (ODI): Oswestry Disability Index; (NDI): Neck Disability Index; (VAS): Visual Analogue Scale.

## Competing interests

The authors declare that they have no competing interests.

## Authors' contributions

CES conceptualized and designed the study, supervised the acquisition of data and data analysis, and drafted the manuscript; JFM, PK, DAW participating in the acquisition of data and participating in revising the manuscript critically for important intellectual content; RB and LL participated in data analysis; DHK conceptualized and designed the study, and drafted the manuscript. All authors read and approved the final manuscript.

## Supplementary Material

Additional file 1 **Appendix 1: ICBG Complications Questionnaire**. Questionnaire used to assess the presence or absence of persistent pain, functional limitation, and cosmesis at the ICBG site. Click here for file
