# Supplementary material for: Prospective evaluation of chronic pain associated with posterior autologous iliac crest bone graft harvest and its effect on postoperative outcome
Source: Health Qual Life Outcomes. 2009 May 29;7:49. doi: 10.1186/1477-7525-7-49 (PMC2693524; doi:10.1186/1477-7525-7-49)
Supplement: Additional file 1 — Appendix 1: ICBG Complications Questionnaire. Questionnaire used to assess the presence or absence of persistent pain, functional limitation, and cosmesis at the ICBG site. [file 1477-7525-7-49-S1.doc]

**Appendix 1: ICBG Complications Questionnaire**

Please answer the following questions regarding the bone graft part of your spine surgery (we are just asking about pain and symptoms from where the bone graft was taken and not about pain from the neck or back surgery itself):

1. The bone graft was more painful than the actual surgery itself:

🞎 Yes

🞎 No

2. Are you bother by how the bone graft harvest scar looks?

🞎 Yes

🞎 No

3. Do you have numbness or abnormal sensation around your bone graft harvest scar?

🞎 Yes

🞎 No

4. If you have numbness or abnormal sensation, does it bother you?

🞎 Yes

🞎 No

5. Does pain from where the bone graft was taken cause you:

Difficulty walking?

🞎 Yes

🞎 No

Difficulty with your job?

🞎 Yes

🞎 No

Difficulty with your recreational activities?

🞎 Yes

🞎 No

Difficulty with your household chores?

🞎 Yes

🞎 No

Difficulty with sexual activity?

🞎 Yes

🞎 No

Irritation from clothing?

🞎 Yes

🞎 No
